# *Diospyros kaki* fruit extract produces antiarthritic and antinociceptive effects in rats with complete Freund's adjuvant‐induced arthritis

**DOI:** 10.1002/fsn3.4418

**Published:** 2024-08-20

**Authors:** Fatemeh Forouzanfar, Motahareh Mirdoosti, Maryam Akaberi, Ramin Rezaee, Seyed‐Alireza Esmaeili, Ehsan Saburi, Hanie Mahaki

**Affiliations:** ^1^ Medical Toxicology Research Center, School of Medicine Mashhad University of Medical Sciences Mashhad Iran; ^2^ Department of Pharmacognosy, School of Pharmacy Mashhad University of Medical Sciences Mashhad Iran; ^3^ Applied Biomedical Research Center Mashhad University of Medical Sciences Mashhad Iran; ^4^ Immunology Research Center Mashhad University of Medical Sciences Mashhad Iran; ^5^ Medical Genetics and Molecular Medicine Department, Faculty of Medicine Mashhad University of Medical Sciences Mashhad Iran; ^6^ Vascular and Endovascular Surgery Research Center Mashhad University of Medical Sciences Mashhad Iran

**Keywords:** *Diospyros kaki*, herbal medicine, mechanical allodynia, natural products, persimmon, thermal hyperalgesia

## Abstract

Current treatments for rheumatoid arthritis produce untoward effects; thus, considerable effort has been made to recognize effective herbal medicines against the condition. In the present study, the therapeutic effect of *Diospyros kaki* fruit hydroalcoholic extract (DFHE) on complete Freund's adjuvant (CFA)‐induced arthritis in rats was investigated. The extract was characterized using liquid chromatography–electrospray mass spectrometry (LC‐ESIMS). Male Wistar rats were grouped as follows (eight rats in each): control, CFA, CFA + indomethacin (5 mg/kg), CFA + DFHE (50 mg/kg), and CFA + DFHE (100 mg/kg). Paw volume, mechanical allodynia, thermal hyperalgesia, and arthritis score were evaluated. Serum levels of malondialdehyde (MDA), thiol groups, tumor necrosis factor‐alpha (TNF‐α), as well as glutathione peroxidase (GPx) and superoxide dismutase (SOD) activities were evaluated. Carotenoids were found to be the major components of DFHE. Administration of DFHE (100 mg/kg) significantly decreased arthritis score, paw volume, and thermal hyperalgesia, and improved mechanical allodynia. MDA and TNF‐α levels were decreased while thiol levels and SOD and GPx activities were increased in DFHE‐treated groups compared to the CFA group. These results suggest that *D. kaki* extract caused an improvement in clinical signs of rheumatoid arthritis symptoms possibly through suppression of oxidative stress and inflammation.

## Graphical abstract

*Diospyros kaki* fruit hydroalcoholic extract reduced arthritis score and pain and alleviated inflammation and oxidative stress status.

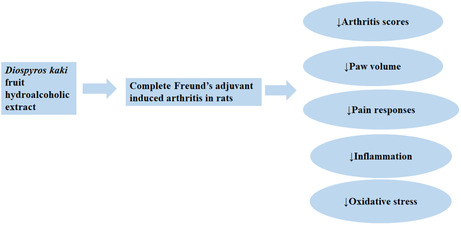

## 1 INTRODUCTION

Rheumatoid arthritis (RA) is a prevalent immune‐mediated illness and its main symptom is inflammatory arthritis typified by symmetric, polyarticular pain, and swelling usually in the hands and feet joints. On the other hand, extraarticular symptoms and a multitude of concomitant disorders are linked to RA as a systemic illness (Gravallese & Firestein, 2023). The global incidence rate of RA is increasing continuously, reaching to age‐standardized incidence rate of 13 per 100,000 people in 2019 (Cai et al., 2023).

The fact that women make up 75% of RA patients suggests the role hormones play in the etiology of the illness. This condition, which is marked by joint stiffness and swelling, frequently in a symmetrical pattern on both sides of the body, is also thought to be exacerbated by stress and smoking. The objectives of RA patient care are to reduce disability, limit discomfort, postpone disease onset, and enhance quality of life (Khanna et al., 2007). The treatment of RA depends on the severity of disease activity; however, treatment mainly includes glucocorticoids, biological disease‐modifying antirheumatic agents like methotrexate, and nonsteroid anti‐inflammatory drugs, all of which produce side effects and require constant monitoring (Fraenkel et al., 2021; Mian et al., 2019). Therefore, attempts to enhance the treatment of RA are of great value.

Oxidative stress is a pathogenic characteristic of RA, and free radicals, which are essential secondary mediators in the RA inflammatory and immunological cellular responses, are indirectly linked to joint destruction. Increased oxidative stress makes T cells resistant to many stimuli, such as those that promote proliferation or death, and it may prolong the aberrant immune response. Besides, free radicals can directly damage joint cartilage by targeting its proteoglycan and preventing its formation (Quiñonez‐Flores et al., 2016). It has long been known that plants have medicinal qualities. People have been using herbal medicine to treat a wide range of illnesses for generations. On the other hand, the exact exploitation of plant‐active chemicals has been the foundation for the development of the pharmaceutical industry during the past century. This increase has produced many herbal natural products and their analogs, which are utilized in clinics to treat diseases like cancer, diabetes, and neurodegenerative disorders. Additionally, plants are a valuable resource for chemical research facilities that promote drug development (Li & Weng, 2017). Recent studies have used herbal products as novel treatments or as adjuvant treatments for chronic illnesses like RA (Wang et al., 2021). Persimmon (*Diospyros kaki* L.) belongs to the family Ebenaceae and its fruit is popular in East Asian countries, particularly in Japan, South Korea, and China (Direito et al., 2021). The health‐promoting properties of persimmon include anticancer (Direito et al., 2017), analgesic (Bawazeer & Rauf, 2021), antiaging (Yokozawa et al., 2011), anti‐inflammatory (Direito et al., 2020), hepatoprotective (Ma et al., 2007), antioxidant (Forouzanfar et al., 2016), and antidiabetic effects (Lee et al., 2006). Antioxidants such as carotenoids, phenolic compounds, and vitamins are abundant in persimmon, and the aforementioned health effects have been linked to their presence (Chen et al., 2008; Direito et al., 2021; Grygorieva et al., 2018).

The current study was conducted to examine the effect of persimmon fruit extract on RA caused by complete Freund's adjuvant (CFA) in rats.

## 2 MATERIALS AND METHODS

### 2.1 Preparation of *D. kaki* hydroalcoholic extract

Fresh fruits of *D. kaki* were purchased from the local market in Mashhad, Iran. *Diospyros kaki* whole‐fruit hydroalcoholic extract (DFHE) (70%) was prepared by a maceration procedure as described previously (Rakhshandeh et al., 2021). The yield was nearly 27% w/w.

### 2.2 Liquid chromatography–electrospray mass spectrometry (LC‐ESIMS) analysis

To elucidate the metabolic profile of the extract, HPLC‐ESIMS analysis was performed. The compounds in the sample were separated by LC technique using an optimized chromatography method, and identified by an ESIMS detector. The concentration of the sample was 10 mg/mL in dimethyl sulfoxide (DMSO). The separation condition was as follows:

Solvent A: water and formic acid 0.1%; Solvent B: methanol and formic acid 0.1%; gradient profile: 20% Solvent B isocratically for 1 min, 20%–100% Solvent B for 40 min; 100% Solvent B in 10 min; solvent flow rate: 0.4 mL/min; column temperature: 25°C; and sample injection volume: 20 μL.

### 2.3 MZmine data preprocessing

The raw MS data files were converted into MzXML files using ProteoWizard Mass Convert software, and then, they were processed using MZmine 3.0 (Schmid et al., 2023). The parameters were set according to our previous study (Zanganeh et al., 2024).

### 2.4 Animals

To perform this study, 25 male Wistar rats weighing 200–250 g were obtained from the animal house of Mashhad University of Medical Sciences (MUMS), Mashhad, Iran. The rats were kept in plexiglass cages under standard circumstances including the day–night light schedule of 12–12 h, temperature of 25°C, and ad libitum available food and water.

### 2.5 Establishment of rheumatoid arthritis (RA) animal model and study protocol

RA was induced by subcutaneous injection of 0.1 mL CFA (each mL contains 1 mg of heat‐killed and dried *Mycobacterium tuberculosis*, 0.85 mL paraffin oil, and 0.15 mL of mannide; Sigma) into the hind paw of rats on day 0 (Forouzanfar et al., 2023).

To perform the experiment, the rats were randomly classified into five groups, each containing eight rats.

1. Control group subcutaneously received 0.1 mL of normal saline in the right hind paw on day 0.
2. CFA group.
3. The indomethacin group was treated with oral indomethacin (5 mg/kg/day, daily) for 21 days.
4. Low‐dose DFHE group received CFA on day 0 and was treated with DFHE 50 mg/kg (oral, daily) for 21 days.
5. High‐dose DFHE group received CFA on day 0 and was treated with DFHE 100 mg/kg (oral, daily) for 21 days.

CFA (0.1 mL) was administrated subcutaneously in the right hind paw on day 0.

### 2.6 Drug administration

*Diospyros kaki* extract was dissolved in saline with 1% dimethyl sulfoxide (DMSO) and administered orally. Behavioral exams, measurement of arthritis score, and paw volume were performed on days 0, 7, 14, and 21 post‐CFA injection. At the end of pain measurement tests, animals were deeply anesthetized with 100 mg/kg ketamine and 10 mg/kg xylazine (i.p.), and blood samples were collected. After blood serums were separated by centrifuge, the isolated serum was collected to investigate oxidative stress status.

### 2.7 Paw volume

The caliper was used to measure the paw's thickness. The rats were restrained, and the diameter of each paw was measured from the distance between all of the pads on the dorsal surface of the hind paw to the plantar surface on the front of the paw. To maintain consistency, the same qualified assessor who was skilled in the particular measurement method carried out each examination (Forouzanfar et al., 2023).

### 2.8 Thermal hyperalgesia

The hot plate was used to assess thermal hyperalgesia. The rats were acclimatized to the examination room for 10 min prior to the beginning. Animals were separately placed on metal hot plate with a temperature of 55°C. When the animal pulls or licks the paw in response to heat, it is recorded and considered as an indicator of pain threshold. To avoid damage, a threshold of 15 seconds was considered (Forouzanfar et al., 2023).

### 2.9 Mechanical allodynia

Mechanical allodynia was quantified using an elevated box with equal dimensions of 30 cm, with a floor of metal wires. First, the rats were placed on the wire mesh floor separated allowing free access to all place preference compartments for 15 min. von Frey filaments (Bioseb, USA) in scores of 2, 4, 6, 8, 10, 15, 26, and 60 g were applied to the paw in ascending order. Stimuli with the same filament were tested five times, and if the rat responded with withdrawal three times, the test result was considered positive. A force of 60 g was considered as the cut‐off threshold to avoid damage (Rakhshandeh et al., 2022).

### 2.10 Rheumatoid arthritis (RA) score

The clinical scoring system (arthritis score) was evaluated by a visual scoring system of the clinical signs and symptoms on a scale of 0–4 per limb (Forouzanfar et al., 2022): 0 = no difference; 1 = digit swelling or mild erythema; 2 = moderate swelling and significant erythema; 3 = severe swelling and erythema spreading to ankle; and 4 = incapability to bend the ankle due to severe inflammation.

### 2.11 Oxidative stress status evaluation

Serum levels of thiol and malondialdehyde (MDA), as well as superoxide dismutase (SOD) activity, were assessed as reported before (Khorrami et al., 2019). The activity of glutathione peroxidase (GPx) was quantified using a commercial GPX assay kit (ZellBio, German).

### 2.12 Inflammation marker status evaluation

Serum tumor necrosis factor‐α (TNF‐α) levels were determined using commercial ELISA kits (Karmania Pars Gene Company, Kerman, Iran).

### 2.13 Statistical analysis

For analyzing the data, GraphPad Prism (version 6.0) was utilized. All quantitative data are shown as mean ± SEM. The results of the behavioral tests of different groups were compared by two‐way analysis of variance (ANOVA), followed by Bonferroni's test. To statistically compare oxidative stress markers data of different groups, a one‐way ANOVA test followed by Tukey's test was employed. *p*‐Values were considered significant at <.05.

## 3 RESULTS

### 3.1 *Diospyros kaki* fruit hydroalcoholic extract (DFHE) characterization

**FIGURE 1:**
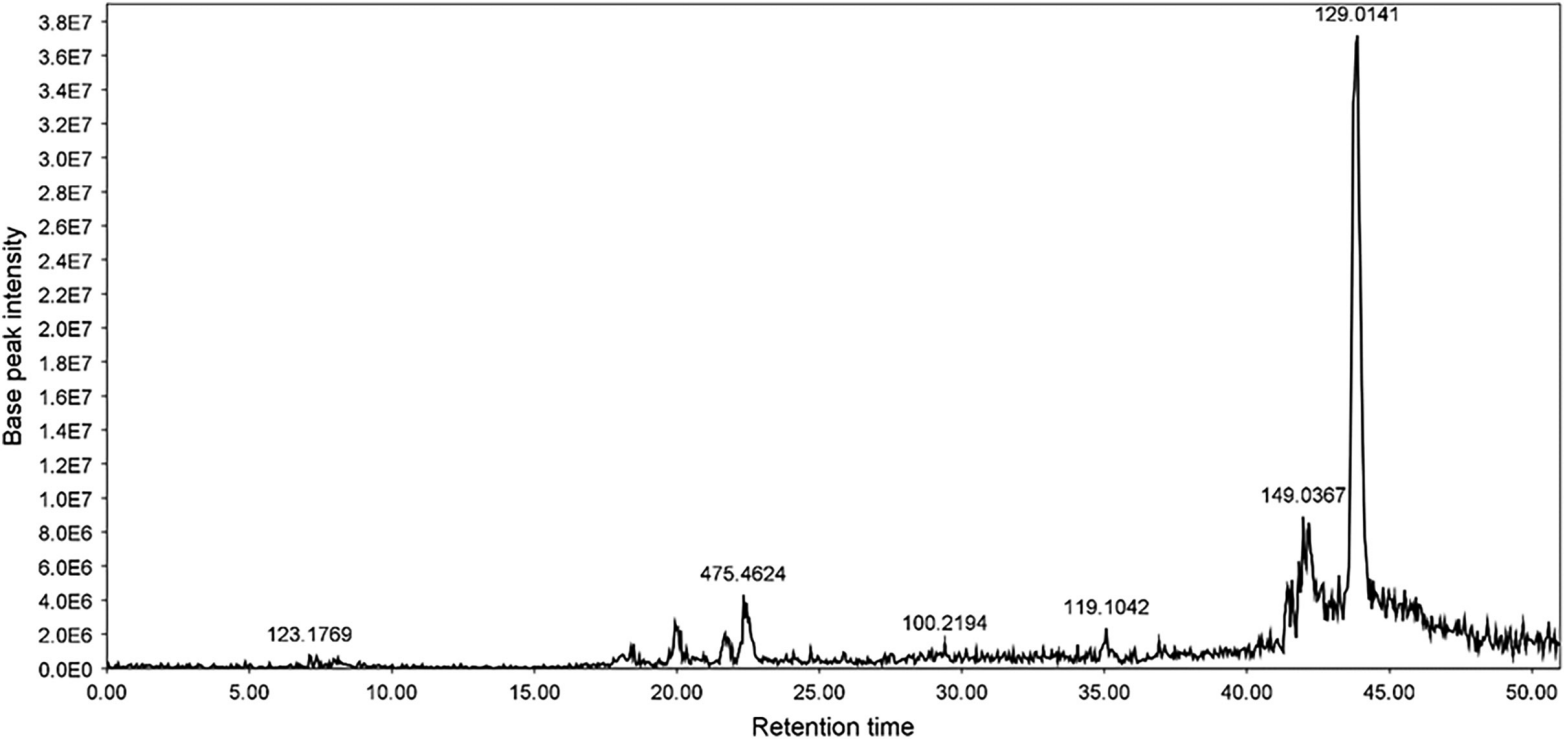
LC‐ESIMS chromatogram of the 70% aq. Ethanol extract of *Diospyros kaki* fruit.

Figure 1 shows the LC‐ESIMS chromatogram of DFHE and Table 1 presents the identified components; we found that carotenoids were the major components of DFHE.

**TABLE 1:** The tentatively identified compounds in the 70% ethanol extract of *Diospyros kaki* fruit hydroalcoholic extract (DFHE).

No.	Retention time	[M + H]^+^	Compound	Tentative identity	References
1	18.38	248.9000	5,6,8‐Trimethoxy‐3‐methyl‐1‐naphthalenol	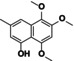	Kim et al. (2021)
2	41.47	537.3688	Carotene	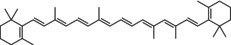	Zhou et al. (2011)
3	41.60	568.6500	Cryptoxanthin epoxide	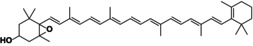	Zhou et al. (2011)
4	41.60	568.6500	Cryptoflavin	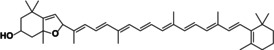	Zhou et al. (2011)
5	43.82	139.0115	2‐Methoxy‐1,4‐benzoquinone	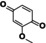	Gaudemer et al. (1985)

### 3.2 Effect of DFHE on RA score

**FIGURE 2:**
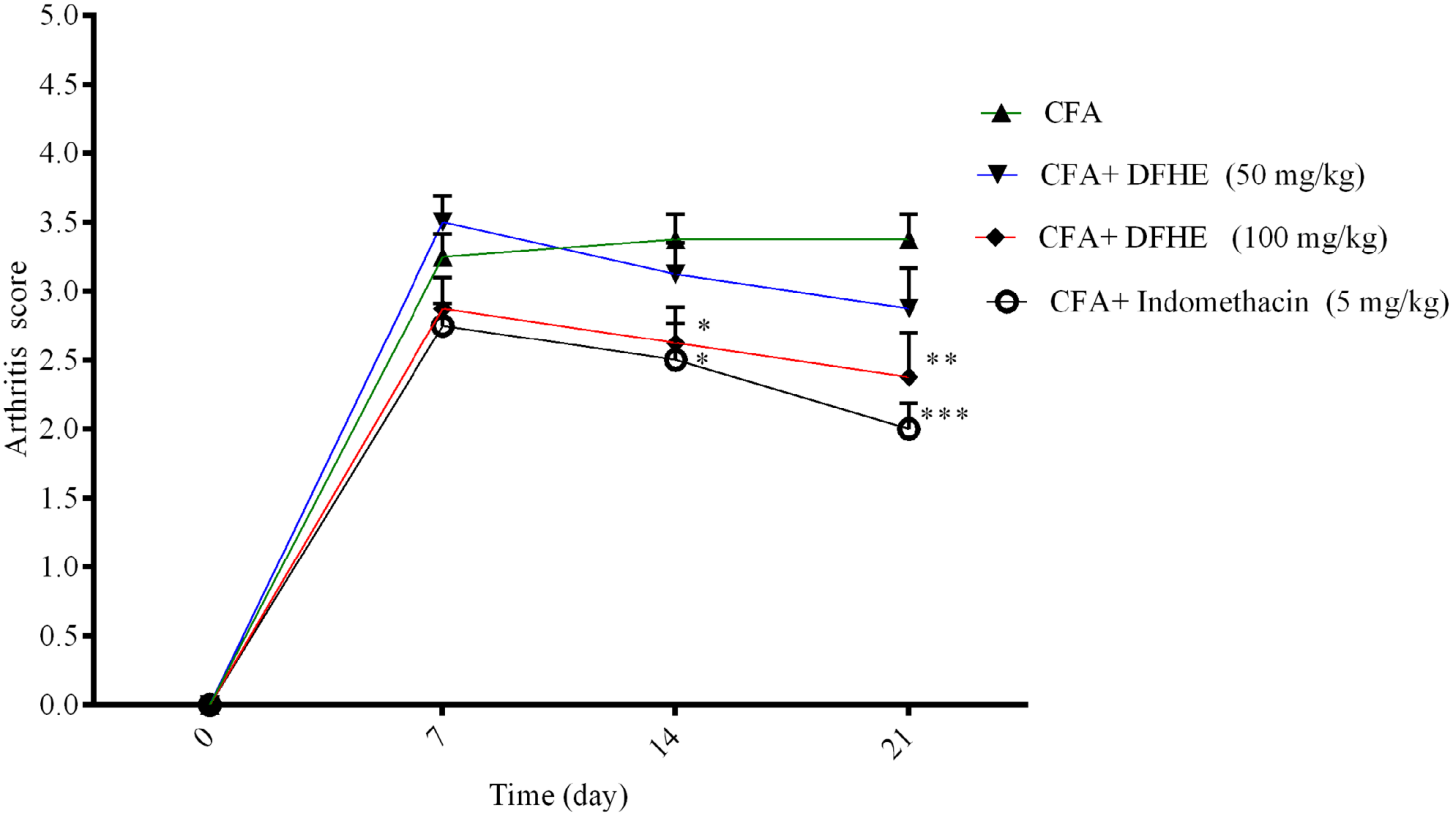
Effect of *Diospyros kaki* fruit hydroalcoholic extract (DFHE) on arthritis score in rats with Freund's complete adjuvant (CFA)‐induced arthritis. Data are shown as mean ± SEM (*n* = 8). *p < .05, **p < .01, and ***p < .001 versus the CFA group.

The RA score was recorded on days 0, 7, 14, and 21 for each group (Figure 2). On day 0, the RA score was 0 for all groups while on days 14 and 21, the score was significantly lower in indomethacin‐ and DFHE (100 mg/kg)‐treated groups compared to the CFA group (*p* < .05, and *p* < .01 respectively).

### 3.3 The effect of DFHE on paw volume

**FIGURE 3:**
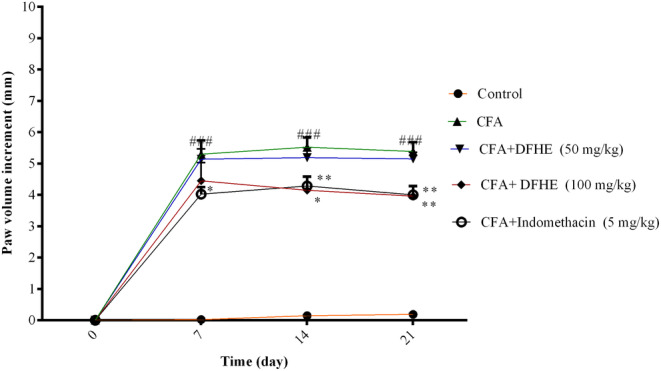
Effect of *Diospyros kaki* fruit hydroalcoholic extract (DFHE) on paw volume increment in rats with Freund's complete adjuvant (CFA)‐induced arthritis. Data are shown as mean ± SEM (*n* = 8). *p < .05 and **p < .01 versus the CFA group. ^###^ *p* < .001 versus the control group.

In arthritic rats, the paw volume was significantly higher on days 7, 14, and 21 of the study versus the control rats (*p* < .001). Treating rats with indomethacin caused a decrease in paw volume versus the CFA‐treated rats on days 7, 14, and 21 of the study (*p* < .05, *p* < .05, and *p* < .01 respectively). DFHE (100 mg/kg) also caused a decrease in paw volume on days 14 and 21 versus the CFA‐treated rats (for both cases, *p* < .01) (Figure 3).

### 3.4 Effect of DFHE on thermal hyperalgesia

**FIGURE 4:**
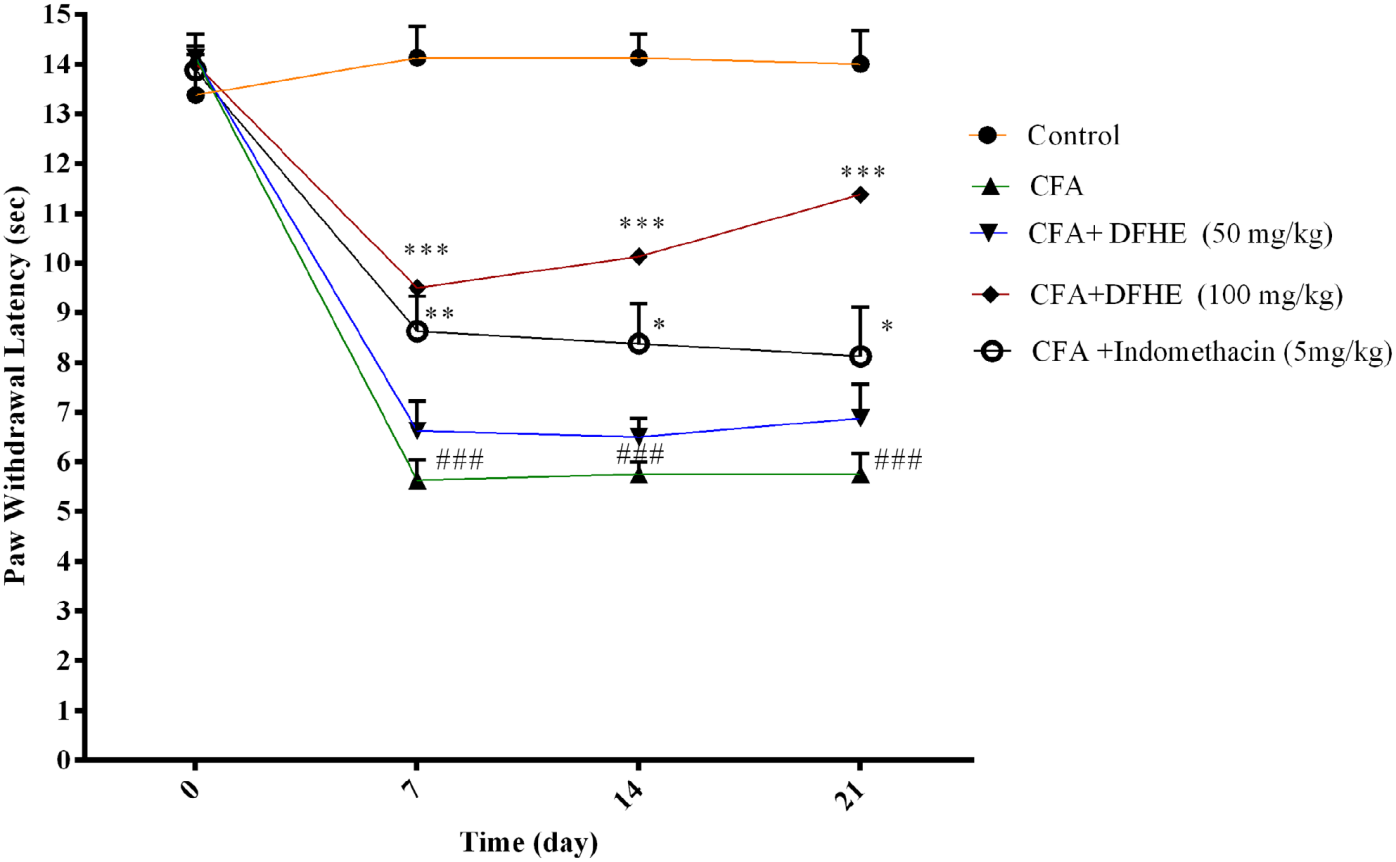
Effect of *Diospyros kaki* fruit hydroalcoholic extract (DFHE) on paw withdrawal latency in rats with Freund's complete adjuvant (CFA)‐induced arthritis. Data are shown as mean ± SEM (*n* = 8). *p < .05, **p < .01, and ***p < .001 versus the CFA group. ^###^ *p* < .001 versus the control group.

The paw withdrawal latency was lower in the CFA group versus the control group on days 7, 14, and 21 (for all cases, *p* < .001). Indomethacin caused an increase in paw withdrawal latency on days 7, 14, and 21 (*p* < .01, *p* < .05, and *p* < .05, respectively). However, DFHE (100 mg/kg) significantly increased the paw withdrawal latency on days 7, 14, and 21 versus the CFA‐treated rats (for all cases, *p* < .001). (Figure 4).

### 3.5 Effect of DFHE on mechanical allodynia

**FIGURE 5:**
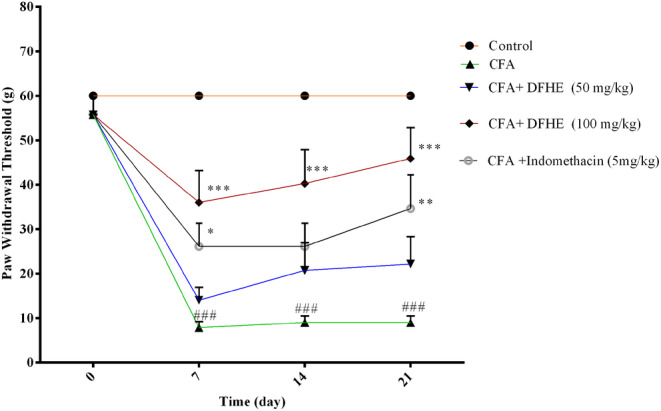
Effect of *Diospyros kaki* fruit hydroalcoholic extract (DFHE) on paw withdrawal threshold in rats with Freund's complete adjuvant (CFA)‐induced arthritis. Data are shown as mean ± SEM (*n* = 8). *p < .05, **p < .01, and ***p < .001 versus the CFA group. ^###^ *p* < .001 versus the control group.

In the CFA group, rats displayed lower paw withdrawal thresholds on days 7, 14, and 21 of the study versus the control rats (for all cases, *p* < .001). Oral administration of DFHE increased the paw withdrawal threshold at the dose of 100 mg/kg on days 7, 14, and 21 days of the study versus the CFA group (for all cases, *p* < .001). Oral administration of indomethacin also enhanced the paw withdrawal threshold on days 7 and 21 days post‐CFA injection versus the CFA‐treated rats (*p* < .05, and *p* < .01, respectively) (Figure 5).

### 3.6 Effect of DFHE on oxidative stress markers

Mean serum MDA level was higher in the CFA‐treated rats versus the control rats (*p* < .001); however, it was significantly lower in the indomethacin‐ and DFHE (100 mg/kg)‐treated groups (for both cases, *p* < .001) compared to the CFA‐treated rats (Table 2). The serum level of thiol was significantly lower in the CFA‐treated rats than in the control rats (*p* < .001). Treatment with DFHE (50 and 100 mg/kg) and indomethacin significantly elevated serum thiol levels compared to the CFA‐treated rats (*p* < .01, *p* < .001, and *p* < .01, respectively).

**TABLE 2:** Effect of *Diospyros kaki* fruit hydroalcoholic extract (DFHE) on serum MDA, thiol concentrations, GPx, and SOD activities in rats with Freund's complete adjuvant (CFA)‐induced arthritis.

	MDA (μmol/L)	Thiol (mmol/L)	SOD (U/L)	GPx (U/mL)	TNF‐α (pg/mL)
Control	6.944 ± 0.8823	20.27 ± 1.545	29.67 ± 1.647	236.8 ± 14.56	62.00 ± 4.082
CFA	11.65 ± 0.3852^###^	4.406 ± 0.7023^###^	13.00 ± 0.7303^###^	121.7 ± 9.458^###^	110.8 ± 6.819^###^
CFA + DFHE (50 mg/kg)	9.509 ± 0.6706	11.59 ± 1.017**	14.17 ± 1.108	163.8 ± 13.45	100.2 ± 5.192
CFA + DFHE (100 mg/kg)	7.340 ± 0.6186***	15.04 ± 1.504***	23.17 ± 1.701***	184.8 ± 17.85*	77.83 ± 5.326 **
CFA + Indomethacin (5 mg/kg)	6.699 ± 0.6094***	11.85 ± 0.9162**	21.83 ± 1.014***	190.8 ± 6.247**	74.83 ± 6.745**

*Note*: Values are expressed as mean ± SEM for six animals. *p < .05, **p < .01, and ***p < .001 versus the CFA group. ^###^ *p* < .001 versus the control group.

In arthritic rats, there were significant reductions in serum GPx activity than the control rats (*p* < .001); treatment with indomethacin or DFHE (100 mg/kg) increased GPx activity versus the CFA‐treated rats (*p* < .05, and *p* < .01, respectively) (Table 2).

Serum SOD activity was markedly lower in the CFA‐treated rats compared to the control rats (*p* < .001). Indomethacin or DFHE (100 mg/kg) administration effectively enhanced serum SOD activity in RA rats (for both cases, *p* < .001) (Table 2).

### 3.7 Effect of DFHE on TNF‐α level

Serum TNF‐α levels were markedly higher in the CFA‐treated rats compared to the control rats (*p* < .001). Indomethacin or DFHE (100 mg/kg) administration effectively reduced serum TNF‐α levels in RA rats (for both cases, *p* < .001) (Table 2).

## 4 DISCUSSION

In the current study, the administration of DFHE to a rat model of CFA‐induced RA reduced pain symptoms as well as arthritis scores and alleviated oxidative stress status. CFA is used to produce in vivo models of RA (Forouzanfar et al., 2023); CFA is mycobacteria, killed by heat, and emulsified by paraffin oil. Paraffin oil cannot be metabolized and causes persistent immune system exposure to antigens. It also facilitates the transportation of antigens through lymph nodes and other organs. Injecting CFA into rat footpad causes a long‐lasting local inflammatory reaction, granuloma formation, and induction of the immune system to constantly produce inflammatory cytokines in the systemic circulation (Billiau & Matthys, 2001). The cornerstone of RA symptoms is excessive pain (Vergne‐Salle et al., 2020); when CFA‐induced arthritis is established in rats, the footpad is swollen, tender, warmer, and more sensitive to pain. Pain is generally caused by any stimulus that can potentially provoke tissue damage, and generally, nociceptors are activated by diverse types of energy (mechanical, thermal, etc.). In the case of settled inflammation, pain occurs with nonpainful stimuli (allodynia), or is perceived as excessively extreme compared to the intensity of the stimulus (hyperalgesia) (Coutaux et al., 2005). Despite advancements in inflammation‐suppressive medications, RA discomfort is still a significant issue. Pain and inflammation are intimately related because they affect central pain processing in addition to causing acute peripheral sensitization in the joint (McWilliams & Walsh, 2017). Early in the course of RA, there is an increase in central pain, which may not go away even after synovitis has been suppressed. Central sensitization may be caused by inflammatory processes that occur locally in the joint as well as throughout the body through the circulation of cytokines and other neuromodulatory substances (McWilliams & Walsh, 2017). Long‐term nociceptive input can alter how the brain interprets pain, and nociceptive input increases when nearby peripheral nerves in the joint become more sensitive. Numerous substances that might sensitize peripheral nerves are produced by synovitis, including bioactive lipids, kinins, neuropeptides (such as calcitonin gene‐related peptide, or CGRP), cytokines (such as TNF‐α, IL‐1, and IL‐6), and neurotrophins (such as nerve growth factor, or NGF). By producing cytokines like IL‐1, immune cells in the CNS directly contribute to the development of central sensitization. Moreover, systemic aspects of inflammation are linked to RA (McWilliams & Walsh, 2017). Given that persistent inflammation may cause the blood–brain barrier to become weakened, circulating cytokines may be able to enter the central nervous system. Further study in animal models suggests a causal connection between enhanced cerebral pain processing and synovitis. Rats with inflammatory arthritis have central sensitization, and their behavior, electrophysiology, and histology all show altered spinal and supraspinal pain processing, often even before the disease's outward symptoms manifest (McWilliams & Walsh, 2017).

In the current study, the rats injected with CFA exhibited a lower threshold of response to mechanical and thermal stimuli determined by hot plate and allodynia tests due to inflamed paws. However, oral administration of DFHE in 100 mg/kg dosage improved RA symptoms such as paw volume, thermal hyperalgesia, mechanical allodynia, and arthritis score. A hot plate test is an easy and quick way to identify thermal hyperalgesia. The animal is settled on a limited hot surface (50–56°C) and the time lag that the animal shakes, jumps, or licks the hind paw has an inverse relationship with pain intensity (Langford & Mogil, 2008). In this study, DFHE significantly increased hot plate latencies.

Pain thresholds can be measured in humans and animals by applying probes with varied surface areas and varying forces. von Frey filament is likely the most widely used method in pressure sensitivity. The technique involves applying flexible filaments with different degrees of stiffness to the area under study. Usually, a force just enough to bend each filament is applied, and the finest filament that triggers a response defines a threshold (Möller et al., 1998). Mechanical allodynia was assessed by examining the response to von Frey filaments which are used to investigate mechanical hypersensitivity (Nicotra et al., 2014). In a higher pain state, the rat withdraws the paw to a lower threshold. Our findings showed that DFHE could alleviate CFA‐induced pain, showing the analgesic effect of *D. kaki* extract. Direito and colleagues also showed that persimmon fruit extract attenuates macroscopic and radiographic signs of inflammation in collagen‐induced arthritis in Wistar rats (Direito et al., 2020).

Reactive oxygen species (ROS) are necessary in a physiological setting to keep the redox state of the cell in balance. Nevertheless, ROS overproduction can harm cellular components including proteins and nucleic acids as well as the lipids in cell membranes. An imbalance between antioxidants and oxidants, favoring oxidants—known as oxidative stress—leads to molecular damage (Quiñonez‐Flores et al., 2016).

In blood and synovial fluid from RA patients, disruption in antioxidant systems and increased levels of lipid peroxidation were observed. The presence of nitrous type II collagen peptide, modified low‐density lipoprotein, oxidized IgG, and cartilage peroxidation products in the blood and urine of RA patients provide indirect evidence that ROS contributes to ligament degeneration (Phull et al., 2018). Besides, proinflammatory cytokines are produced in response to ROS generation. Moreover, the inflammation process which involves host immune cells producing significant levels of ROS through the NADPH oxidase enzyme pathway exacerbates oxidative stress (García‐Sánchez et al., 2020).

The pathogenesis of RA has been linked to lipid peroxidation. Polyunsaturated fatty acids (PUFAs) are oxidized during lipid peroxidation to create lipid peroxyl radicals which then cause more oxidation of PUFAs that can harm cell membranes. Lipid hydroperoxides and conjugated dienes are produced when lipids, PUFAs, are oxidatively damaged. These compounds then break down to produce a variety of byproducts such as volatile hydrocarbons, hydroxyalkenals, alkanols, and MDA (Vasanthi et al., 2009).

Vitamins A and C, as well as reduced glutathione, are part of the nonenzymatic antioxidant defense. On the other hand, enzyme‐based antioxidants include catalase, GPx, glutathione‐S‐transferase (GST), SOD, and glutathione reductase (GR) (Mateen et al., 2016). According to earlier research, oxidative stress and RA are linked to higher serum levels of lipid peroxidation, lower levels of glutathione, and lower activities of SOD and catalase (Ren et al., 2019; Shan et al., 2019). Our findings corroborated the above‐mentioned evidence, showing that administration of DFHE reduced oxidative stress markers in CFA‐induced arthritis rats.

Tumor necrosis factor alpha (TNF‐α) is a proinflammatory cytokine secreted by cells such as monocytes and macrophages in response to inflammation. It is a proinflammatory immune response's signature cytokine (Zamri & De Vries, 2020). It has been determined that TNF‐α medication is an effective treatment approach for RA (He et al., 2022). Administration of DFHE and indomethacin reduced TNF‐α levels in CFA‐induced arthritis rats.

As a medication with several uses, indomethacin is a nonsteroidal anti‐inflammatory medicine (NSAID). It prevents prostaglandins from being synthesized, which are important mediators of inflammation, fever, and pain, and are mostly produced by cyclooxygenase enzymes. The FDA has approved indomethacin for the treatment of acute pain, gouty arthritis, ankylosing spondylitis, osteoarthritis, bursitis, and patent ductus arteriosus (Munjal & Allam, 2023). Despite its notable effectiveness in alleviating symptoms of certain arthritic conditions, it has been demonstrated that indomethacin does not alter the disease's progression (Lucas, 2016).

In our present study, indomethacin reduced pain behavior, paw volume, arthritic score, TNF‐α level, and oxidative stress markers in CFA‐induced arthritis rats. Carbohydrates, carotenoids, organic acids, phenolics, and tannins are the main constituents of persimmon fruit. The pulp of persimmon is a source of carbohydrates (glucose, fructose, and sucrose), phenolic acids including ferulic, *p*‐coumaric, and gallic acids, phenolics such as catechin, epicatechin, and condensed proanthocyanidins, and carotenoids mainly *p*‐cryptoxanthin, zeaxanthin, lycopene, carotene, and lutein. Compared to the skin of the fruit, the pulp has lower amounts of carotenoids. The most abundant carotenoids in the pulp are β‐carotene, α‐carotene, β‐cryptoxanthin, and zeaxanthin (Cui et al., 2023; Gaudemer et al., 1985; Jia et al., 2023; Kim et al., 2021; Ye et al., 2022). In one study, rats with collagen‐induced arthritis were treated with persimmon extracts to assess its anti‐inflammatory properties. The persimmon extracts significantly reduced the amount of edema and radiological changes in the bone, soft tissue swelling, and localized loss of bone, along with the growth of osteophytes in the tibiotarsal joint that was linked to collagen‐induced arthritis (Direito et al., 2020).

The defatted chloroform extract of *D. kaki* (200 mg/kg) showed a protective effect in the carrageenan‐induced paw edema model. Also, the extract diminished paw edema in the histamine‐induced paw edema model (Bawazeer & Rauf, 2021). A previous study confirmed that among acetone, ethanol, methanol, and water, the more effective in extracting antioxidant ingredients from persimmon ingredients was obtained using ethanol (Jang et al., 2010).

In conclusion, *D. kaki* extract causes a significant improvement in behavioral signs, serum oxidative stress, and inflammation markers in the CFA‐induced rat model of arthritis.

## AUTHOR CONTRIBUTIONS

**Fatemeh Forouzanfar:** Formal analysis (equal); investigation (equal); methodology (equal); supervision (equal); writing – original draft (equal); writing – review and editing (equal). **Motahareh Mirdoosti:** Investigation (equal); writing – original draft (equal). **Maryam Akaberi:** Formal analysis (equal); investigation (equal); writing – original draft (equal). **Ramin Rezaee:** Investigation (equal); writing – review and editing (equal). **Seyed‐Alireza Esmaeili:** Investigation (equal); writing – review and editing (equal). **Ehsan Saburi:** Investigation (equal); methodology (equal); writing – review and editing (equal). **Hanie Mahaki:** Investigation (equal); writing – original draft (equal).

## FUNDING INFORMATION

This work was supported by the Vice Chancellor for Research and Technology, MUMS, Mashhad, Iran (Grant No. 4001732).

## CONFLICT OF INTEREST STATEMENT

The authors declare no conflicts of interest.

## ETHICS STATEMENT

All procedures were approved by the Ethics Committee on Animal Research of MUMS (Ethics approval No. IR.MUMS.MEDICAL.REC.1401.135).

## Data Availability

The datasets of the current study are available from the corresponding author on request.
