# EXPRESSION OF CONCERN: *Diospyros Kaki* Fruit Extract Produces Antiarthritic and Antinociceptive Effects in Rats With Complete Freund's Adjuvant‐Induced Arthritis

**DOI:** 10.1002/fsn3.70115

**Published:** 2025-04-07

**EXPRESSION OF CONCERN**: F. Forouzanfar, M. Mirdoosti, M. Akaberi, R. Rezaee, S.‐A. Esmaeili, E. Saburi and H. Mahaki, “ *Diospyros Kaki* Fruit Extract Produces Antiarthritic and Antinociceptive Effects in Rats With Complete Freund's Adjuvant‐Induced Arthritis,” *Food Science & Nutrition* 12, no. 10 (2024): 8084–8092, https://doi.org/10.1002/fsn3.4418.

This Expression of Concern is for the above article, published online on 20 August 2024 in Wiley Online Library (wileyonlinelibrary.com), and has been issued by agreement between the journal Editor‐in‐Chief, Y. Martin Lo; and Wiley Periodicals LLC. The Expression of Concern has been agreed due to implausible SEM values presented in Figures 2, 3, 4 and 5, and also within Table 2. The authors provided an explanation along with some supporting data; however, this was not deemed satisfactory to remove doubt. Furthermore, flaws in the methodology have been identified, specifically the decision to study just two doses of DFHE. Therefore, the journal has decided to issue an Expression of Concern to inform and alert the readers.

